# Reconstruction of massive full‐thickness abdominal wall defect: successful treatment with nonabsorbable mesh, negative pressure wound therapy, and split‐skin grafting

**DOI:** 10.1002/ccr3.649

**Published:** 2016-09-07

**Authors:** Dogu Aydin, Ida Felbo Paulsen, Vibeke Egerup Bentzen, Sami Asadzadeh, Lisbet Rosenkrantz Hölmich

**Affiliations:** ^1^ Department of Plastic and Reconstructive SurgeryHerlev‐Gentofte HospitalUniversity of CopenhagenCopenhagenDenmark; ^2^ Department of Gastrointestinal SurgeryHerlev‐Gentofte HospitalUniversity of CopenhagenCopenhagenDenmark

## Abstract

We demonstrate that it is possible to use a nonabsorbable mesh for abdominal wall reconstruction after total wound rupture and successfully split‐skin graft directly on the mesh. Sufficient granulation tissue formation prior to skin grafting was obtained with long‐term use of negative pressure wound therapy (NPWT).

## Introduction

Abdominal wall defects most commonly result from wound rupture as a complication to surgery. Predisposing factors are both extrinsic and intrinsic; contaminated wounds probably is the most important extrinsic factor along with surgical technique, while obesity, malnutrition, diabetes, and other comorbidities are responsible for intrinsic predisposing factors 1, 2. A complete abdominal wall defect is life‐threatening, and if the patient survives, it will be with a mutilating functional and cosmetic result impacting quality of life, mobility, physical capacity, and ability to cough, urinate, and defecate 1, 2. Handling of the totally open abdomen is highly challenging and costly. The first priority is to get closure of the abdominal contents, whereas skin closure is secondary. The use of abdominal negative pressure wound therapy (NWPT) with plastic draping over the abdominal content has become standard of care in most centers 2, 3 as a temporary mean to get the abdominal walls retracted toward the midline, handling swollen soft tissue as well as edematic inner organs due to heavy infection, trauma, etc. 4, 5, 6. Reconstruction of the abdominal wall will normally entail use of mesh, and in the infected setting, acellular dermal matrix (ADM) has gained increasing usage, as ingrowth and adherence occur also in contaminated and even infected wounds 6. Nonabsorbable meshes in the infected patient will result in unsuccessful healing due to biofilm and insufficient ingrowth of tissue, and this is a common cause of loss of the implant 7. However, synthetic mesh used in combination with NPWT providing temporary coverage can be successful probably due to reduced infection and enhanced granulation tissue formation over the implant 8, 9. We report a rare patient case with a massive abdominal wall defect, who was treated in this manner and with subsequent split‐skin grafting.

## Case Report

**Figure 1:**
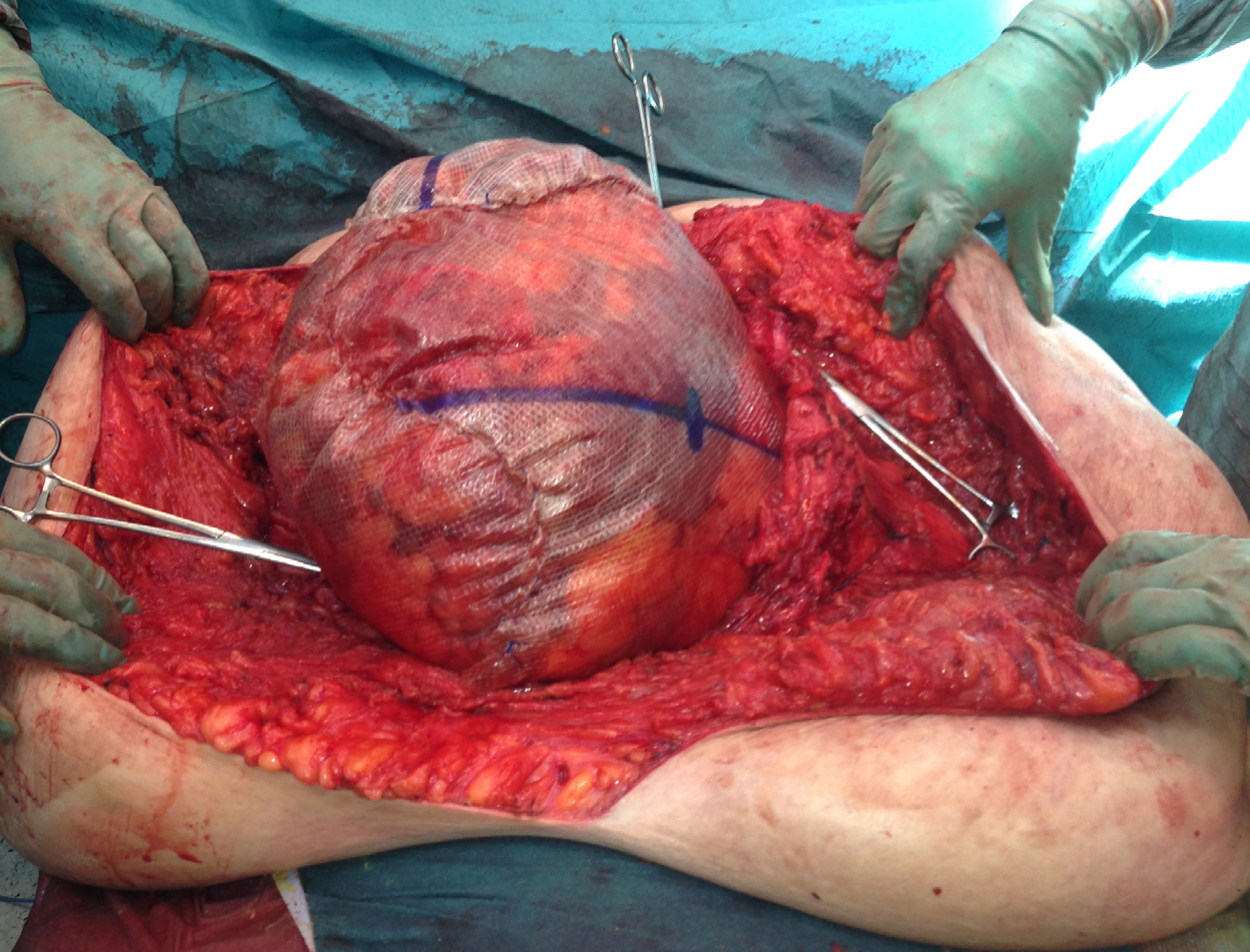
Perioperative photograph with three pieces of synthetic mesh sewn together and to the facial edges (shown with peans) to substitute the abdominal wall.

In May 2014, a 51‐year‐old morbidly obese woman (BMI 48), suffering from non‐insulin‐dependent diabetes, presented at the emergency department with a 20‐year‐old incisional hernia that had become incarcerated. At admittance, the abdomen was described with horizontal and vertical scars from former surgery and a discolored midline hernia measuring 30 × 50 cm. Acute CT of the abdomen demonstrated small bowel obstruction, and an immediate explorative laparotomy was performed via the vertical scar. Perioperatively, the abdominal wall defect measured 15 × 10 cm, and the ventricle, colon, and small intestine were encapsulated in a subcutaneous cavity. The lower 150 cm of the small intestine was cyanotic and torqued around the mesentery; detorquing resulted in revitalization. The intra‐abdominal cavity was found shrunken and too small to contain the intestinal organs. Consequently, the fascia was split proximally and distally of the hernia port; however, the abdominal wall could not be closed without the use of three pieces of nonabsorbable mesh each measuring 35 × 35 cm (Fig. 1). The intra‐abdominal organs were enclosed in the new cavity, and the skin was closed above the cavity.

**Figure 2:**
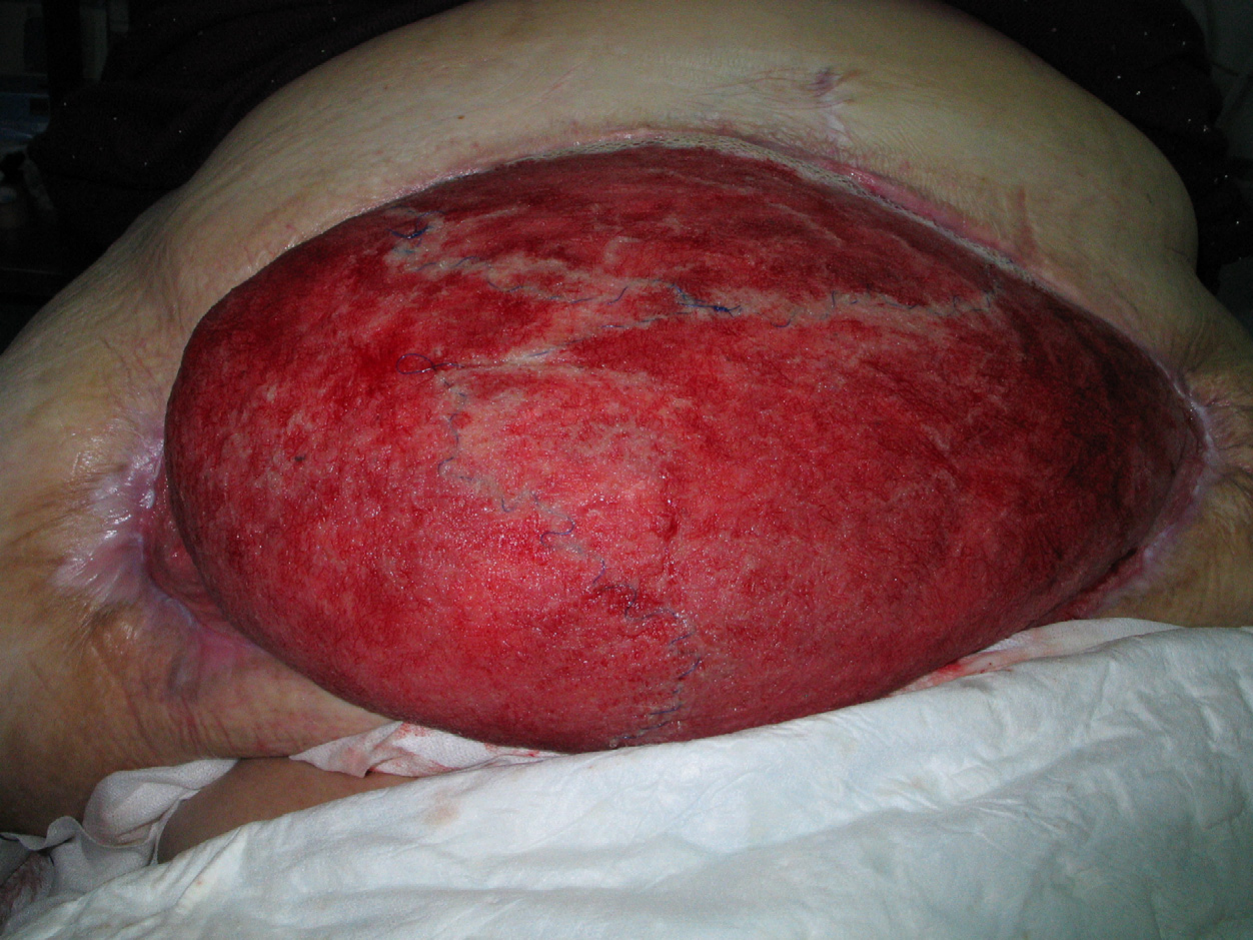
After eight months of VAC treatment and just prior to split‐skin transplantation. Note the mesh is almost invisible and the abdominal skin has reattached to the lateral aspects of the truncus.

**Figure 3:**
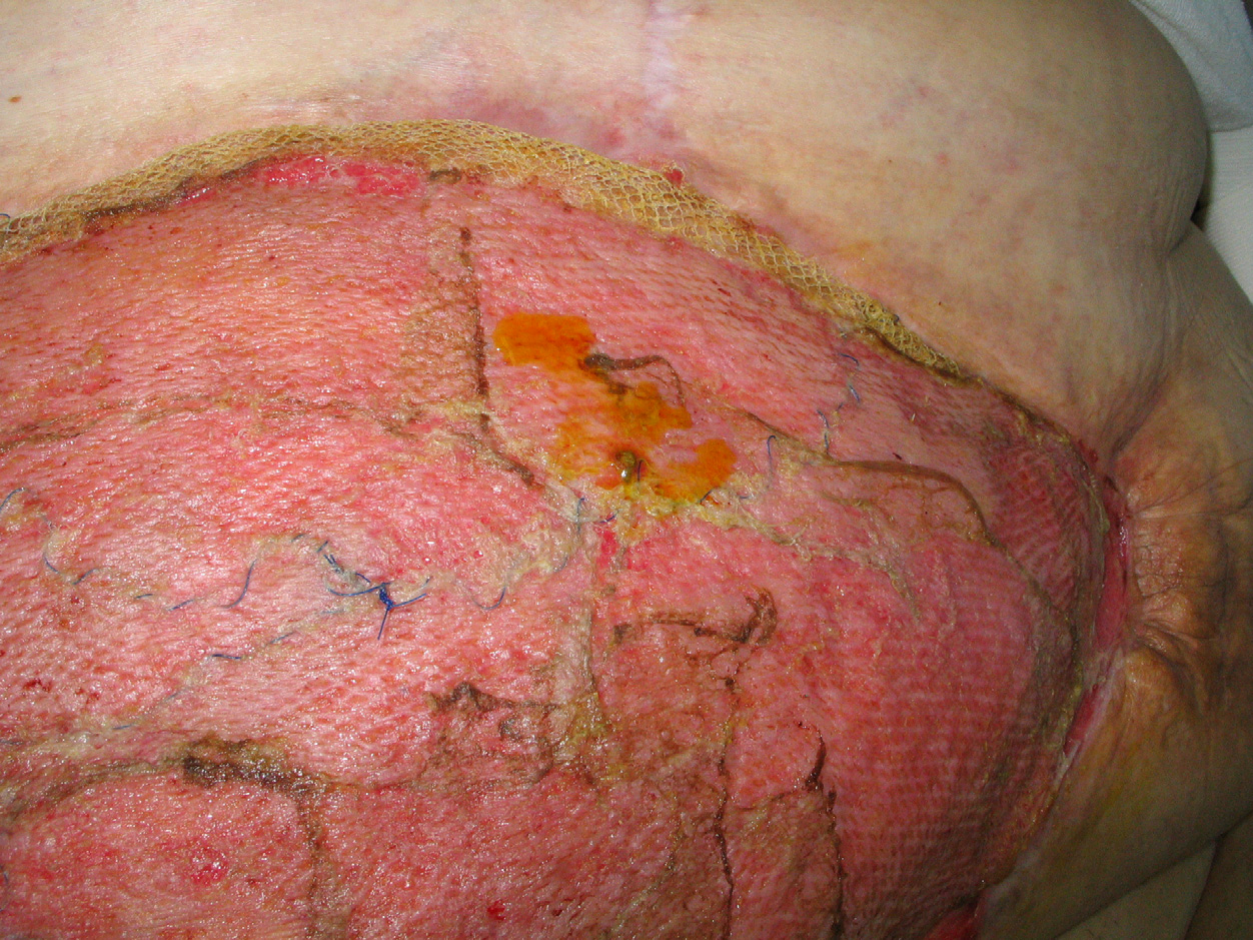
One week after split‐skin transplantation. Note good graft take, folding of the mesh in the upper aspect of the defect and yellow secretion from what turned out to be a high small bowel fistula with low output.

**Figure 4:**
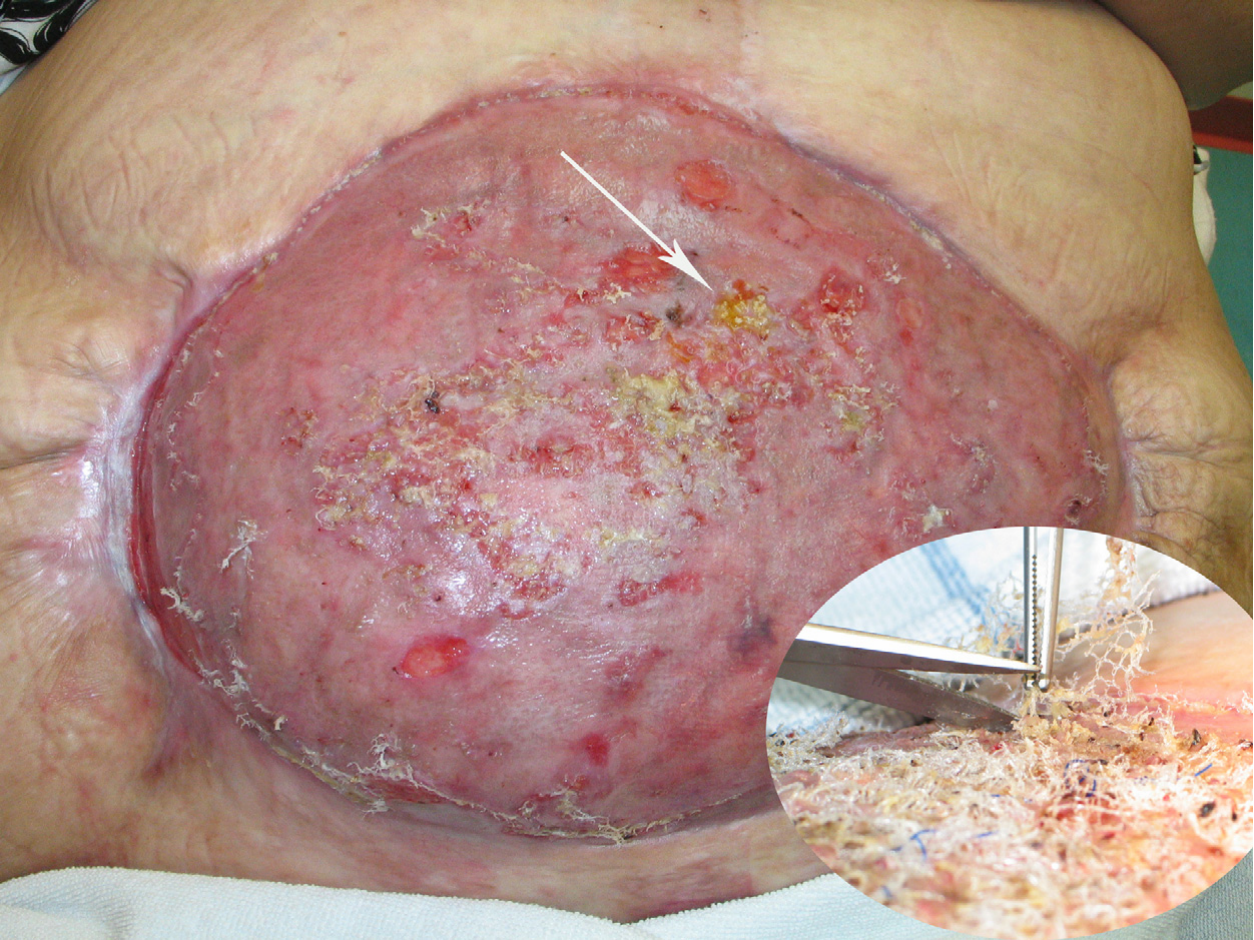
Two months after split‐skin transplantation. The inset photograph six weeks after transplantation. Huge amount of mesh eroded through the transplanted skin and cut off successively.

In the following days, the patient gradually developed necrosis of the skin covering the mesh. The wound was revised and necrotized tissue excised leaving a 40 × 50 cm defect with the underlying mesh being intact. Thereafter, the patient was treated with NPWT and surgically revised or cleansed and dressed every other or third day for a period of 8 months, aiming for granulation tissue formation upon the mesh. General anesthesia was used for the first 3 months, and thereafter, wound care was provided with the use of intravenous sedatives and pain medication, but no anesthesia. After another 2 months, wound dressings were performed in the ward without medication, and 5.5 months after the hernia operation, the patient was discharged and wound dressings were performed in the outpatient clinic. One specific nurse was dedicated to the task. After a total of 8 months, the mesh was covered with granulation tissue without infection (Fig. 2) and split‐skin grafts were harvested from both thighs and transplanted to the defect. The patient afterwards developed a low‐output fistula from the small intestine, treated conservatively (Fig. 3). The fistula sealed 6 months postoperatively. After immediate take of the transplanted skin, the mesh started to erode the overlying vital transplant and this problem worsened as the patient was mobilized (Fig. 4). The mesh was very stiff, and wrinkles damaged the overlying skin. A long period of careful and meticulous removal of the exposed mesh was performed bedside or later in the outpatient clinic with tiny bits or threads being removed successively. Now, two years after, a thread can occasionally be removed, but more and more seldom.

**Figure 5:**
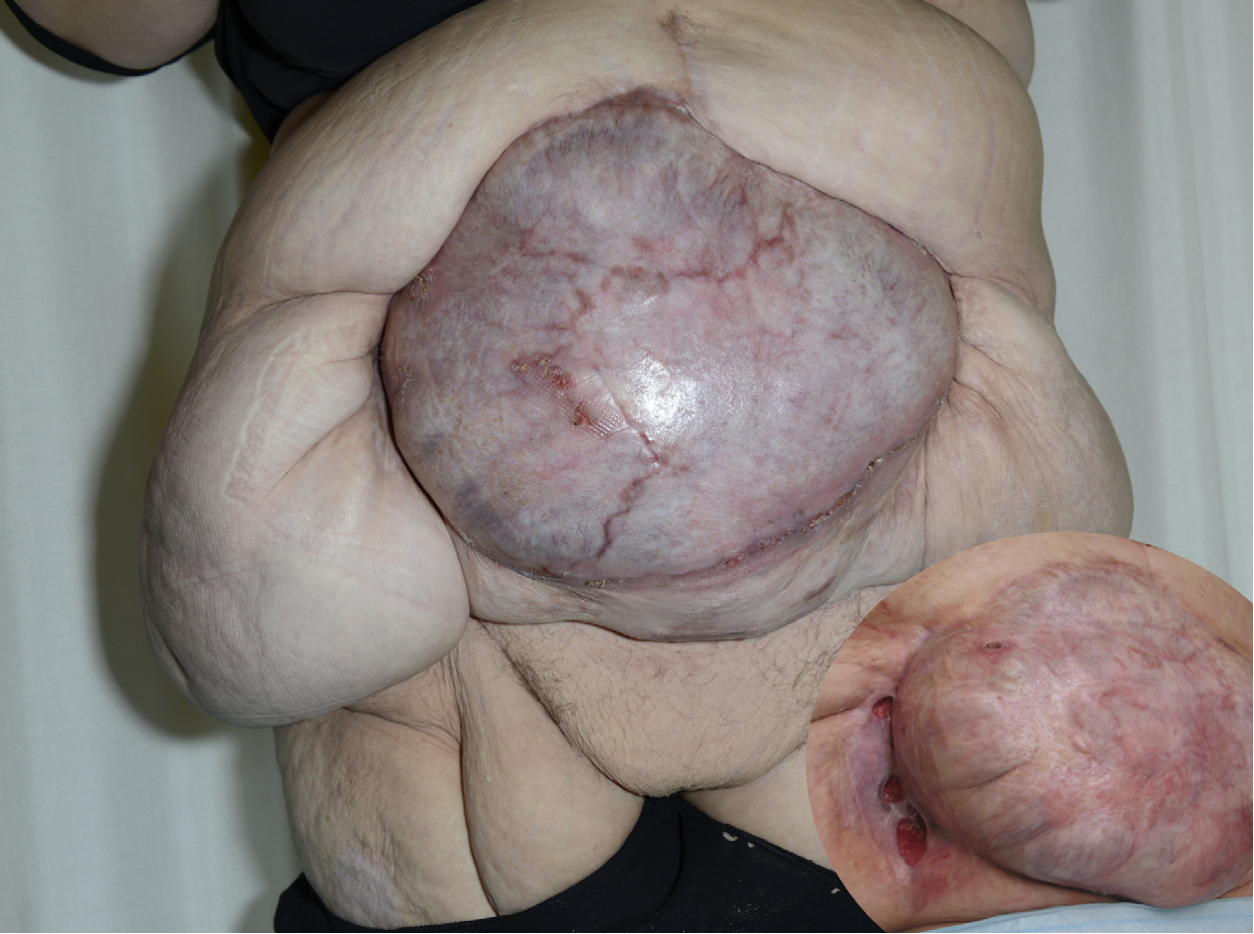
Nine months after skin transplantation. The patient is almost healed. Inset: 28 months after the hernia operation and 20 months after the skin transplantation. A chronic skin defect in the right lateral groove, presumably due to the heavy weight of the lateral role and mechanical collision.

Today, our patient has begun her everyday life working 14 h per week. She has a small skin defect in the right lateral area where the native skin meets the transplanted area (Fig. 5). The lateral skin area especially in the right side is very heavy, and this in combination with the strong elastic belt she must use to keep the hernia in place creates friction in the wound area. Unfortunately, the patient has gained about 20 kg since discharge from the hospital, which has been counterproductive for this problem. If massive weight loss can be accomplished, reduction of the excess skin and perhaps even hernia repair could be pursued, although the latter would entail considerable risk of intestinal damage.

## Discussion

We presented a difficult case with an abdominal disaster after ileus in a heavily obese woman with a 20‐year history of a large incisional hernia. The abdominal cavity was too small to contain all the intestines, and acute repair with a large synthetic mesh was performed; however, the abundant skin, which had to be mobilized to repair the hernia, died subsequently resulting in a 40 × 50 cm large open wound with only the mesh covering the intestines. After 8 months of treatment with negative pressure wound therapy, we managed to perform split‐skin transplantation on the mesh – a procedure which would generally not be considered feasible. In the contemporary literature, only one study reported a similar technique 2. However, none have previously described the problems with folding of the net and eroding of the skin transplant. The described method is beneficial in patients with great comorbidities as in the present case (obesity and diabetes). The drawback is, of course, its cost and need for long hospitalization and outpatient treatment.

Even though an absorbable mesh would be preferred in contaminated conditions, it would perhaps not be able to provide the needed durability and strength for the abdominal wall function as required in this case, and this is why a synthetic mesh was chosen in the particular case 10, 11. A synthetic mesh is considered stronger, as the biological mesh will relax somewhat over time 10.

On the other hand, a nonabsorbable mesh without coverage has an almost guaranteed risk of biofilm formation and complicated ingrowth 12. Any of the above interventions pose great risk of complications; however, in such a case it is a matter of survival of the patient and both functional and cosmetic issues must have lower priority. In this case, nonabsorbable synthetic mesh was chosen due to its durability and strength, which was highly required in this obese patient with a very large abdominal wall defect. It was combined with NPWT, stimulating an anti‐infectious, granulation tissue‐ and neoangiogenesis‐promoting environment. In addition, the NPWT provided a firm bandage for the patient supporting the abdomen. Due to NPWT, we were along the line able to manage the patient's care on a cost‐effective outpatient basis reducing the length of hospital stay and risk of nosocomial infection. Additionally, the open synthetic mesh allowed granulation tissue formation to grow and cover the surface, finally enabling split‐skin grafting. The erosion of the transplanted skin occurred after initial healing, and the mesh had to be removed piece by piece, but as this was performed successively, fibrous tissue replaced the mesh and the skin defects healed. To our knowledge, this phenomenon has not been reported previously. An important factor in the success of this extremely demanding case was that one single nurse highly experienced in complex wound treatment was dedicated to the task. Not only wound expertise, but also massive psychosocial support must be provided in such cases.

## Conflict of Interest

None declared.

## Consent

Written informed consent was obtained from the patient for publication of this case report and any accompanying images. A copy of the written consent is available for review by the editor of this journal.
